# Ikaros antagonizes DNA binding by STAT5 in pre-B cells

**DOI:** 10.1371/journal.pone.0242211

**Published:** 2020-11-12

**Authors:** Beate Heizmann, Stéphanie Le Gras, Célestine Simand, Patricia Marchal, Susan Chan, Philippe Kastner

**Affiliations:** 1 Institut de Génétique et de Biologie Moléculaire et Cellulaire (IGBMC), Illkirch, France; 2 Institut National de la Santé et de la Recherche Médicale (INSERM), U1258, Illkirch, France; 3 Centre National de la Recherche Scientifique (CNRS), UMR7104, Illkirch, France; 4 Université de Strasbourg, Illkirch, France; 5 Service d’Hématologie, Institut de Cancérologie Strasbourg Europe (ICANS), Strasbourg, France; 6 Faculté de Médecine, Université de Strasbourg, Strasbourg, France; German Cancer Research Center (DKFZ), GERMANY

## Abstract

The *IKZF1* gene, which encodes the Ikaros transcription factor, is frequently deleted or mutated in patients with B-cell precursor acute lymphoblastic leukemias that express oncogenes, like BCR-ABL, which activate the JAK-STAT5 pathway. Ikaros functionally antagonizes the transcriptional programs downstream of IL-7/STAT5 during B cell development, as well as STAT5 activity in leukemic cells. However, the mechanisms by which Ikaros interferes with STAT5 function is unknown. We studied the genomic distribution of Ikaros and STAT5 on chromatin in a murine pre-B cell line, and found that both proteins colocalize on >60% of STAT5 target regions. Strikingly, Ikaros activity leads to widespread loss of STAT5 binding at most of its genomic targets within two hours of Ikaros induction, suggesting a direct mechanism. Ikaros did not alter the level of total or phosphorylated STAT5 proteins, nor did it associate with STAT5. Using sequences from the *Cish*, *Socs2* and *Bcl6* genes that Ikaros and STAT5 target, we show that both proteins bind overlapping sequences at GGAA motifs. Our results demonstrate that Ikaros antagonizes STAT5 DNA binding, in part by competing for common target sequences. Our study has implications for understanding the functions of Ikaros and STAT5 in B cell development and transformation.

## Introduction

Interleukin 7 (IL-7) is crucial for early B cell differentiation in the bone marrow. It is required for pro-B cell survival and the proliferative burst that follows pre-BCR signaling in large pre-B cells. However, IL-7 signaling must be attenuated for subsequent cell cycle exit and immunoglobulin light chain recombination [1]. The mechanism responsible for IL-7 downregulation is thought to rely mainly on cell migration [2]. While pro-B cells are tightly associated with IL-7 producing stromal cells, pre-BCR signaling leads to changes in integrin signaling pathways and cell motility, allowing the cells to move away from IL-7 rich niches. It remains unclear, however, if other mechanisms are deeply involved.

We and others previously reported that the transcription factor Ikaros is absolutely required for the transition between the large pre-B and small pre-B stages [3–5]. Ikaros upregulation in primary large pre-B cells causes gene expression changes that mimic in large part those triggered by IL-7 withdrawal, suggesting that Ikaros antagonizes the transcriptional program downstream of IL-7 and mediated by STAT5 [6]. Ikaros is also an important tumor suppressor in human B-cell precursor acute lymphoblastic leukemias (BCP-ALL) [7–9], with a high frequency of loss-of-function mutations in cases with activated STAT5-dependent oncogenic pathways, like BCR-ABL translocations, CRLF2 amplifications or JAK2 activating mutations [10–12]. These combined observations suggest that Ikaros may be an important negative regulator of IL-7/STAT5 dependent transcriptional programs.

Similar to our study in primary pre-B cells, antagonism between Ikaros and STAT5 was recently reported in murine leukemic pre-B cells expressing constitutively activated STAT5b [13, 14]. These authors showed that Ikaros and STAT5 target a large number of common genomic sites, and observed that IL-7 stimulation of the leukemic cells leads to decreased Ikaros binding at the promoter of the *Cish* gene, a well-known STAT5 target. They hypothesized that Ikaros and STAT5 compete for binding to regulatory elements. Nonetheless it remained unclear if these proteins actually competed for binding, and/or if they acted via other mechanisms. It was also unclear if Ikaros modulates the overall efficiency of STAT5 binding. In this report we addressed these questions using an Ikaros-deficient murine large pre-B cell line [3].

## Methods

### Cell lines and transfection

The BH1-IkER cell line was previously described [3]. It derives from a bone marrow cell culture of a Ikzf1^f/f^ x Mb1-Cre^+^ mouse, where Cre-mediated deletion of the last exon of the *Ikzf1* gene takes place at the pro-B cell stage. BH1 cells are blocked in differentiation at the large pre-B cell stage. The COS cell line was transfected with 10 μg of the empty expression vector pTL2, or expression vectors for the mouse Ikaros-1 isoform or the constitutive mouse STAT5a mutant (derived from Addgene plasmid #83255) [15] using lipofectamine.

### Chromatin immunoprecipitation

The general protocol for ChIP was described previously [16]. After pre-cleaning with Magna ChIP protein A Magnetic Beads (Millipore), cell equivalents (50 x 10^6^) were diluted 2.5x in 0.01% SDS, 1.1% Triton X-100, 1.2 mM EDTA, 16.7 mM Tris-HCl pH 8.1, 167 mM NaCl_2_, and incubated overnight (ON) with anti-Ikaros A3 [5 μg (purified rabbit polyclonal antibody against the C-terminal region of murine Ikaros, made in-house)] or anti-STAT5 [5 μg AF2168 (R&D System) or 5 μg ab7969 (Abcam)]. Protein–DNA complexes were bound to 30 μl Protein A Magnetic Beads for 5–6 h at 4°C and washed 1x with low-salt buffer (20 mM Tris-HCl pH 8.1, 150 mM NaCl_2_, 2 mM EDTA, 1% Triton X-100, 0.1% SDS), high-salt buffer (20 mM Tris-HCl pH 8.1, 500 mM NaCl_2_, 2 mM EDTA, 1% Triton X-100, 0.1% SDS), LiCl buffer (10 mM Tris-HCl pH 8.1, 1 mM EDTA, 1% deoxycholate, 1% NP40, 0.25 M LiCl) and Tris EDTA (10 mM Tris-HCl pH 8, 1 mM EDTA). Samples were eluted, cross-linking was reversed and DNA was purified using the iPure Kit (Diagenode).

### ChIP-sequencing

All ChIP-sequencing (ChIP-seq) experiments were performed concomitantly, using cells cultured in parallel with vehicle (EtOH) or 4OHT. Libraries were prepared according to standard Illumina protocols, and were validated with the Agilent Bioanalyzer. Single 50 bp read sequencing runs were performed on Hiseq 2500. Image analysis and base calling was performed with the Illumina pipeline, and reads were aligned to the mm9 mouse genome with Bowtie v0.12.8 [17] with the following parameters “-m 1—strata—best -y -S -l 40 -p 2”. The numbers of unique reads were respectively 25,730,590, 28,892,264, 28,660,996 and 23,567,116 for the input, STAT5 (-4OHT), STAT5 (+4OHT) and Ikaros samples. For visualization in the UCSC genome browser, Wig files were generated by extending the reads to 200 bp length and the read densities in 25 bp bins were computed. All analyses were performed with unique reads that did not overlap with >5 bp with known short or long interspersed nuclear elements, long terminal repeats and satellites.

Peak calling was performed with MACS v1.4.2 [18] using default parameters except for “-g mm”. Peaks were annotated relative to genomic features using Homer v4.1 [19], with annotations extracted from gtf file downloaded from ensembl v67 [20]. Heatmaps and K-means clustering was done using seqMINER v1.3.3g [21]. Analysis of peak overlaps was performed with the Bioconductor package ChIPpeakAnno [20]. Motif analysis was performed with MEME v4.10 [22], using sequences located +/- 40 nt upstream and downstream of the peak summit.

ChIP-seq data were desposited in the GEO database under the accession number GSE134679.

### ChIP qPCR

ChIP q-PCR analysis was performed with the SYBR Green system (Qiagen) with the following primers: *Cish-184* (F 5'GTCCAAAGCACTAGACGCCTG; R 5'TTCCCGGAAGCCTCATCTT), *Cish+4200* (F 5'TACCCCTTCCAACTCTGACTGAGC; R 5'TTCCCTCCAGGATGTGACTGTG), [23], *Socs2* (F 5'AGAAAGTTCCTTCTGGAGCCTC; R 5'GGTTGCCGGAGGAACAGTC), *Bcl6* (F 5'CCTGGTGTCCGGCCTTTCCTAG; R 5'CTGCTGCGGAGCAATGGTAAAGCC), *Ptma* (F 5'TGGCGACACACAGTCGGAC; R 5'CTCACTTGGCTGTCGAACTA).

### Electrophoretic Mobility Shift Assay (EMSA)

EMSA was performed as described [24] using the probes indicated here or in the figures. *Csn2*: 5'CGAGGGACTTCTTGGAATTAA; BS4: 5'TCAGCTTTTGGGAATGTATTCCCTGTCA. Protein sources were nuclear extracts of COS cells transfected with murine Ikaros-1 isoform, constitutive active murine STAT5a, or the empty pTL2 expression vector (Mock).

### Flow cytometry

The following antibodies were used: anti-CD127-BV421 A7-R34 (Biolegend), anti-STAT5 D206Y (Cell Signaling), anti-pSTAT5 (Tyr694) D47E7 (Cell Signaling) and anti-rabbit AF647 (Jackson Immunoresearch). Intracellular staining was performed using the Foxp3/transcription factor staining buffer set (eBioscience). Cells were analyzed with FACS LSR II and FlowJo software.

### Nuclear extract preparation, immunoprecipitation and Western blot

Nuclear extracts were prepared as described previously [16]. 25 μg nuclear extracts were diluted 3x with 25 mM Tris pH6.7, 1 mM EDTA, 5% Glycerol, 1% NP40 and 25 mM NaCl. The extracts were split in two, diluted to 500 μl with the same buffer, and incubated ON at 4°C with anti-Ikaros A3 (1:100) or anti-STAT5 [1:100; #94205 (Cell Signaling)] antibodies, respectively. After 3h incubation with 30 μl protein A sepharose and three washing steps, the samples and the input were resolved by 8% SDS-PAGE and transferred to nitrocellulose membranes. Antibodies used: anti-Ikaros A3, anti-STAT5 (AF2168), anti-MTA2 (ab8106, Abcam), horseradish peroxidase–conjugated anti-rabbit and anti-mouse antibodies (Jackson Immunoresearch). Blots were revealed with the enhanced-chemiluminescence system.

## Results

### Ikaros and STAT5 target a common set of loci

To determine if Ikaros and STAT5 bind to common target regions in pre-B cells, we analyzed the genomic binding profiles of Ikaros and STAT5 in the IL-7-dependent BH1-Ik1ER cell line, by ChIP-sequencing. The BH1 pre-B cell line was derived from a bone marrow cell culture of a Ikzf1^f/f^ x Mb1-Cre^+^ mouse. In Ikzf1^f/f^ x Mb1-Cre^+^ mice, the last exon of the *Ikzf1* gene was specifically deleted at the pro-B cell stage. BH1-Ik1ER cells carry a fusion protein of the Ikaros-1 full-length isoform and the modified ligand binding domain of the estrogen receptor (Ik1-ER). In BH1-Ik1ER cells, Ikaros is translocated to the nucleus upon induction with the synthetic estrogen receptor ligand 4-hydroxytamoxifen (4OHT) [3]. Ikaros binding was mapped in cells cultured without IL-7 and with 4OHT, and STAT5 binding was evaluated in cells cultured with IL-7 and without 4OHT, to exclude the possible interference of one factor on the binding of the other.

**Fig 1:**
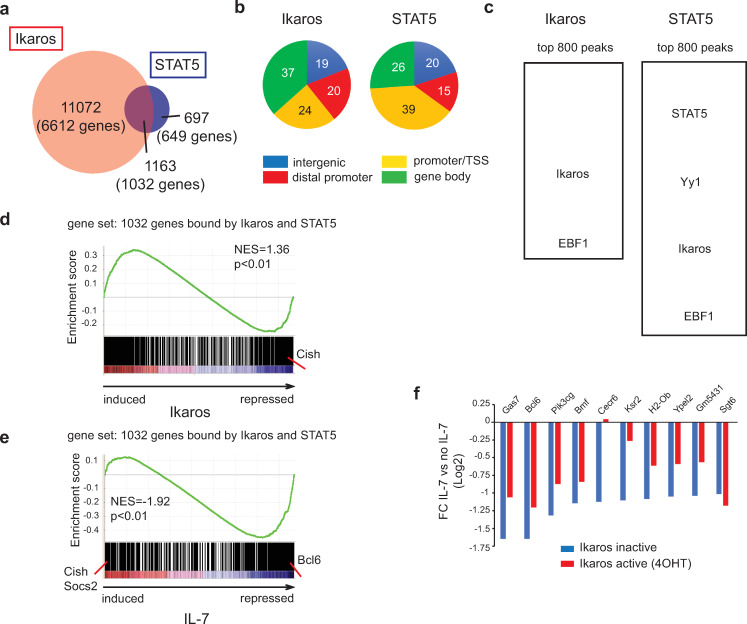
Ikaros and STAT5 DNA binding correlates with Ikaros- and IL-7-dependent gene expression. **(a)** Venn diagram depicting the number and overlap of Ikaros and STAT5 binding peaks. **(b)** Distribution of Ikaros and STAT5 binding peaks on transcriptional start site (TSS) regions (defined as located between -1kb and +100bp of the TSS), gene body (all exons and introns), distal promoter regions (located between -20kb and -1kb of the TSS) and intergenic regions (all other regions). **(c)** Enriched motifs among the Ikaros and STAT5 bound peaks. Enriched motifs were identified with the MEME algorithm within an 80 bp window centered on the peak summit, and significantly enriched motifs are depicted. Motif analysis was performed on the top 800 peaks [ranked by a score corresponding to nb tags x fold enrichment over input x -10log(p-value)]. **(d)** GSEA using as gene set genes bound at common genomic regions by both Ikaros and STAT5, and as the ranked gene list all probesets present on the 430 2.0 array, ranked according to the fold change (FC) of expression between IL-7 deprived cells cultured in the presence or absence of 4OHT (24h). **(e)** GSEA of the same gene set as in (d), where the gene list was ranked according to the FC of expression values measured for cells cultured with or without IL-7 (24h), in the absence of 4OHT. NES: normalized enrichment score. In (d) and (e), the p value is calculated by GSEA on the basis of 100 random permutations of the ranked gene list. **(f)** Comparison of IL-7-dependent repression in the presence and absence of Ikaros. Genes that were bound by both Ikaros and STAT5 at common regions, and repressed by IL-7 more than 2-fold in the absence of 4OHT, were selected. IL-7-dependent FCs (IL-7 vs no IL-7) were calculated for cells cultured in the presence of vehicle (Ikaros inactive) or 4OHT (Ikaros active). In (d), (e) and (f), transcriptome data are from the dataset GSE51350.

We identified 12,235 genomic regions bound by Ikaros and 1860 bound by STAT5 (Fig 1A). Ikaros binding was observed more frequently at sites far from the transcriptional start site (TSS), like gene bodies and distal 5' regions (Fig 1B), while STAT5 binding was detected closer to the TSS (-1kb to +100 bp). The strongest Ikaros peaks (top 800) were enriched for two motifs related to Ikaros binding (GGAAg/a and Ct/aGGa/gA), and the EBF1 motif (Fig 1C). The strongest STAT5 peaks (top 800) were enriched for STAT5 motifs, as expected, but also for motifs of YY1, Ikaros and EBF1, all factors important for early B cell development.

**Fig 2:**
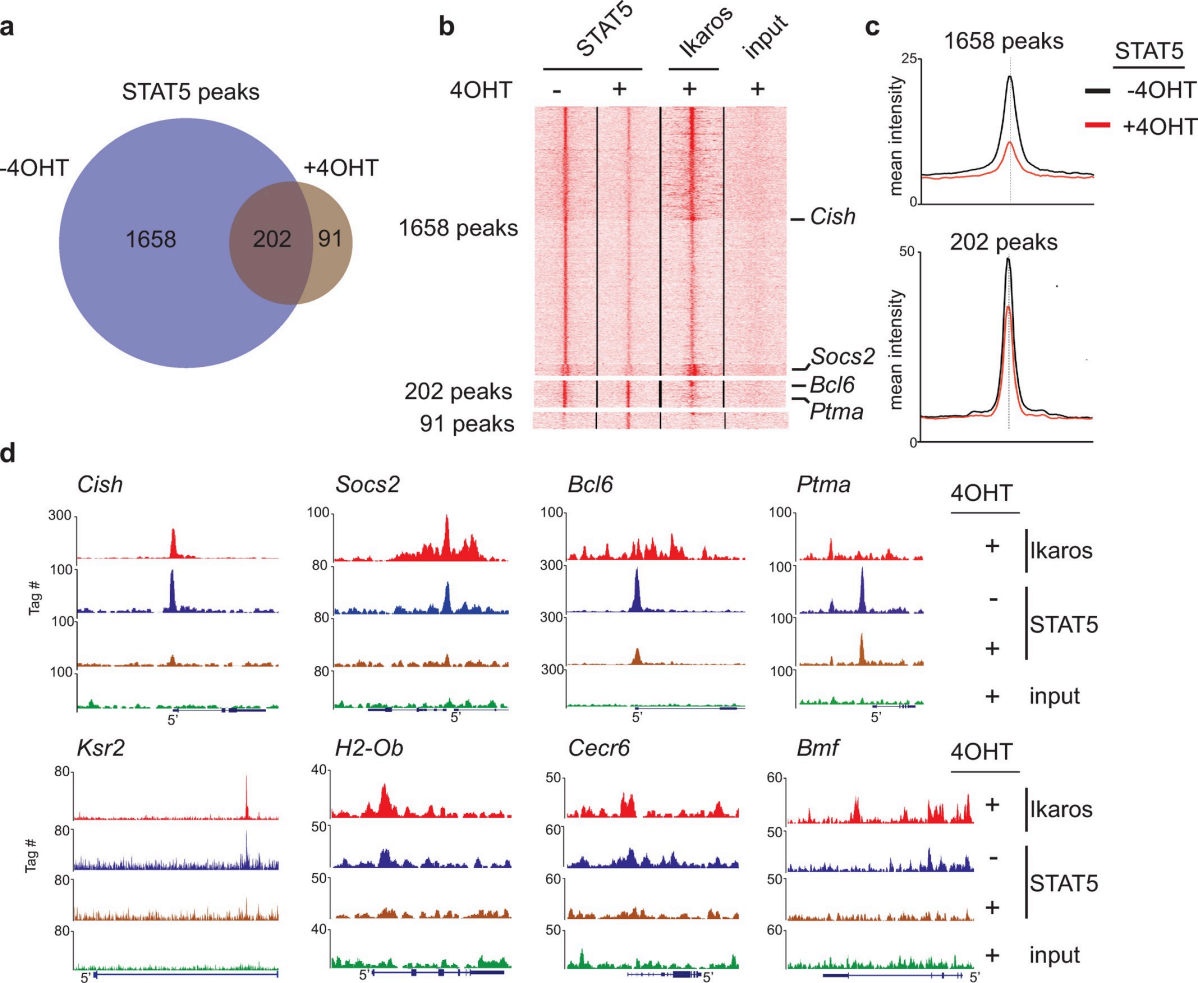
Loss of STAT5 binding upon Ikaros expression at common target genes. **(a)** Venn diagram depicting the size and overlap of the STAT5 bound regions in BH1-IK1ER cells cultured in the absence and presence of 4OHT. **(b)** K-means clustering of the tag densities around the summits of the STAT5 peaks identified in the absence or presence of 4OHT [groups of sites defined in panel (a)]. The position of the peaks illustrated in Fig 2C is indicated. **(c)** Mean intensity profiles for STAT5 for the sets of 1658 and 202 peaks depicted in panels (a) and (b). **(d)** Representative genome browser tracks for 7 genes where STAT5 binding is strongly reduced by Ikaros (*Cish*, *Socs2*, *Bcl6*, *Ksr2*, *H2-Ob*, *Cecr6* and *Bmf*), and one gene (*Ptma*) for which STAT5 binding was not affected.

Nearly two-thirds (1032 genes, 62.5%) of the STAT5-bound regions overlapped with those bound by Ikaros (Fig 1A), suggesting that these factors regulate common target genes. To determine if the genes associated with the overlapping peaks changed expression after Ikaros or STAT5 binding, we analyzed our previous transcriptome data of BH1-Ik1ER cells for Ikaros-regulated (no IL-7, ± 4OHT) and STAT5-regulated (± IL-7, no 4OHT) genes [3]. Gene set enrichment analysis (GSEA) [25] of the 1032 shared target regions revealed a similar enrichment for genes up- or downregulated by Ikaros (Fig 1D), but a strong enrichment for genes downregulated by IL-7/STAT5 (Fig 1E). Among the smaller group of IL-7-induced genes were *Socs2* and *Cish*, two known STAT5-activated genes. Indeed, *Cish* (but not *Socs2*) was strongly downregulated by Ikaros (Fig 1D), suggesting antagonistic regulation. Among the large group of STAT5-repressed genes, *Bcl6* was both strongly downregulated (3-fold) by IL-7/STAT5 (Fig 1E), and displayed a sharp STAT5-binding peak (Fig 2D). The STAT5 repression bias was not observed with genes bound only by STAT5 (S1 Fig).

We then asked if the common target genes repressed by IL-7/STAT5 were affected by Ikaros, and studied 10 genes repressed >2-fold by IL-7 (Fig 1F). Ikaros activity (+4OHT) partially relieved the repression of 8 genes (including *Bcl6*), and completely reversed the repression of one (*Cecr6*), indicating that Ikaros binding antagonizes IL-7/STAT5 repression. In contrast, Ikaros did not affect the expression of common targets activated by IL-7/STAT5 (S2 Fig). These results suggested that Ikaros binding selectively antagonizes STAT5-mediated gene repression.

### Ikaros binding results in widespread loss of STAT5 binding

**Fig 3:**
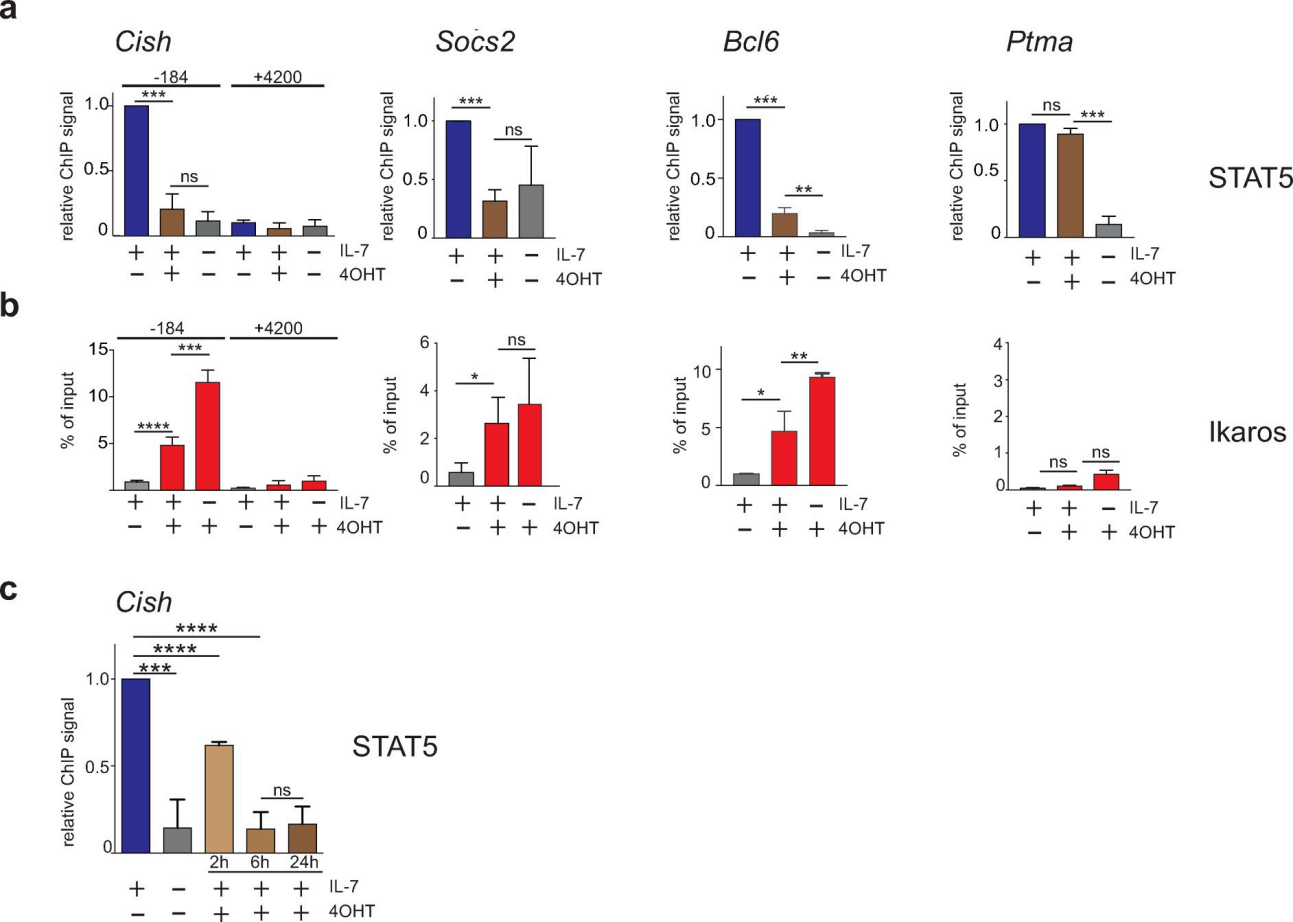
Antagonistic DNA binding by Ikaros and STAT5. **(a)** ChIP-qPCR analysis of STAT5 binding to the same genomic regions as those displayed in Fig 2. Data are from 5 (*Cish*) and 3 (*Socs2*, *Bcl6*, *Ptma*) independent ChIP experiments. The +4200 region of the *Cish* gene is a control region where Ikaros or STAT5 binding was not detected by ChIP-seq. **(b)** ChIP-qPCR analysis of Ikaros binding in the same samples as those used in Fig 2, except for *Cish* for which data are from 4 of the samples. **(c)** STAT5 binding to the -184 bp region of the *Cish* gene in cells treated with 4OHT for various periods of time. Data are from 3 independent ChIP experiments. In (a) and (c), data were normalized to the values obtained in the condition (+IL-7, -4OHT) and statistical analysis was performed with the paired t-test. In (b), statistical analysis was performed with an unpaired t-test. (*) p<0.05; (**) p< 0,01; (***) p< 0,001; (****) p<0,0001; ns: not significant.

To determine if Ikaros binding affects STAT5 binding at common target regions, we evaluated STAT5 binding in BH1-IkER cells after Ikaros induction (IL-7, + 4OHT) by ChIP-seq. Remarkably, Ikaros binding dramatically decreased the number of sites bound by STAT5, from 1860 to 293 (Fig 2A). Only a minority (202) of the STAT5 sites was still observed in the presence of 4OHT. In addition, strong STAT5 peaks, like those seen in the *Cish*, *Socs2*, *Bcl6*, *Ksr2*, *H2-Ob*, *Cecr6* and *Bmf* genes (IL-7, no 4OHT), were barely detectable after Ikaros induction (Fig 2B–2D), though others (eg. at the *Ptma* TSS) were less altered. Indeed, most of the regions with decreased STAT5 binding were clearly bound by Ikaros upon 4OHT treatment (Fig 2B and 2D). Loss of STAT5 binding at common targets was confirmed by ChIP-qPCR (Fig 3A). Furthermore, Ikaros antagonizes STAT5 early, as STAT5 binding is reduced at the *Cish* TSS in 4OHT-treated cells only 2h after Ikaros induction (Fig 3C), suggesting a direct effect. To determine if STAT5 inhibits Ikaros binding, we measured Ikaros binding to common target genes in the presence or absence of IL-7. Ikaros binding increased significantly at *Cish* and *Bcl6* (compare second and third bars) when IL-7 was removed from the media (Fig 3B). A small, but not statistically significant, increase of Ikaros binding was also seen at *Socs2*. These observations suggested that STAT5 can also block Ikaros binding. Collectively, our results indicated that Ikaros and STAT5 bind common target regions in a largely exclusive manner.

### Ikaros activity does not affect STAT5 activation

**Fig 4:**
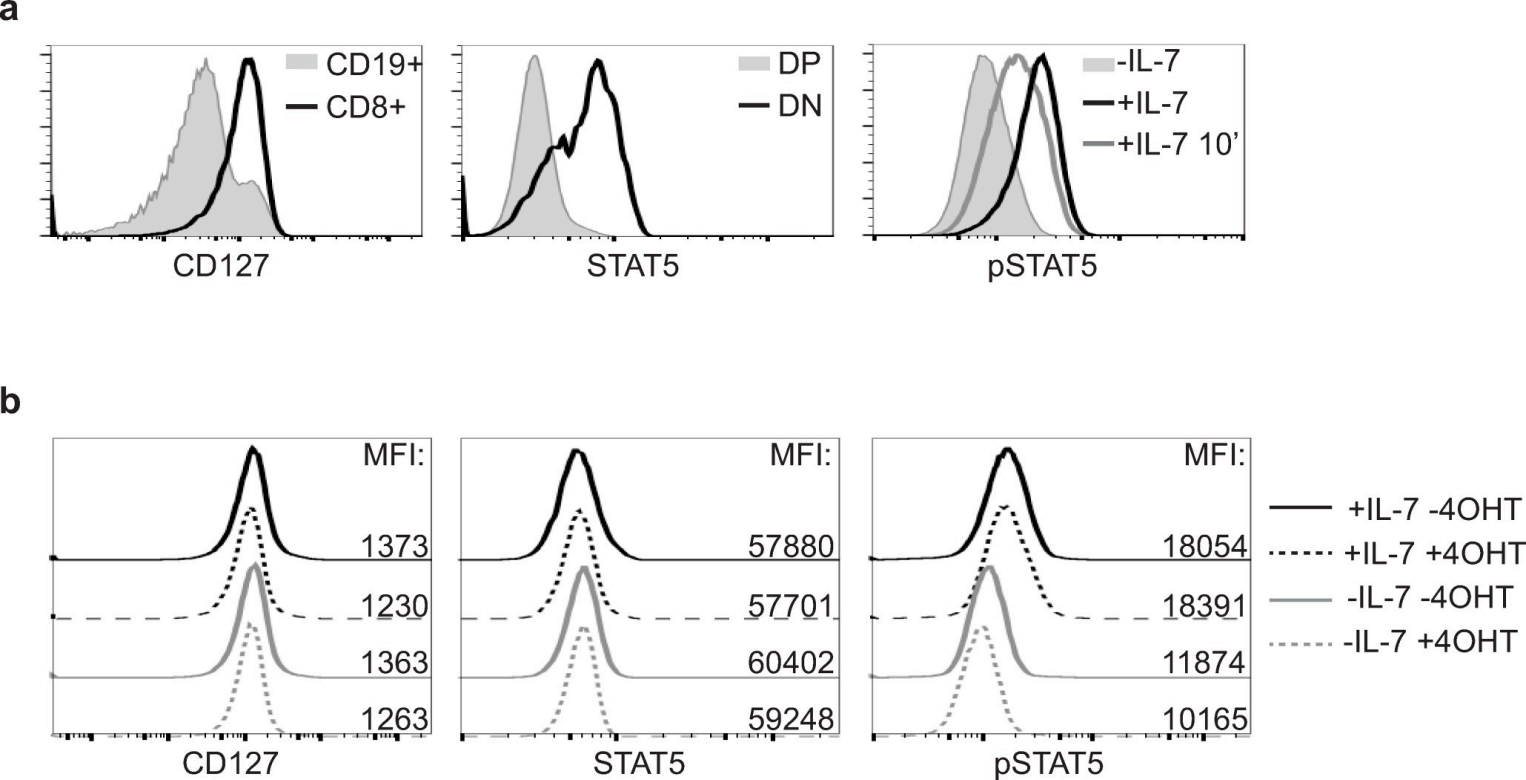
Ikaros does not affect the level of activated STAT5 proteins. **(a)** Controls for the analysis by flow cytometry of the expression of CD127 on splenic CD19^+^ B cells or CD8^+^ T cells (left panel), total STAT5 in double negative (DN) and double positive (DP) thymocytes (middle panel) and pSTAT5 in BH1 cells cultured with or without IL-7, or re-stimulated for 10 min after IL-7 withdrawal (+IL-7 10') (right panel). **(b)** Flow cytometry analysis of BH1-IkER cells upon 24h culture and stained as indicated. Numbers indicate the mean fluorescence intensities (MFI) of the corresponding samples.

To determine if Ikaros induction also affects overall STAT5 protein levels and/or STAT5 phosphorylation, we analyzed the expression of IL-7Rα (CD127), STAT5 and pSTAT5 after 4OHT treatment, by flow cytometry (Fig 4). As controls, we evaluated cells expressing high levels of these proteins (CD127: splenic CD19^+^ B and CD8^+^ T cells; STAT5: CD4^-^CD8^-^ and CD4^+^CD8^+^ thymocytes; pSTAT: BH1-IkER cells cultured with or without IL-7) (Fig 4A). We then analyzed BH1-IkER cells cultured for 24h with or without IL-7, in the absence or presence of 4OHT. These results showed that CD127 and total STAT5 levels increased, while pSTAT5 decreased, after IL-7 was removed from the media, as expected. However, Ikaros induction did not affect the expression of all three proteins regardless of IL-7 status (Fig 4B). Thus, Ikaros does not affect IL-7/STAT5 signaling according to these criteria.

### Ikaros and STAT5 do not interact

**Fig 5:**
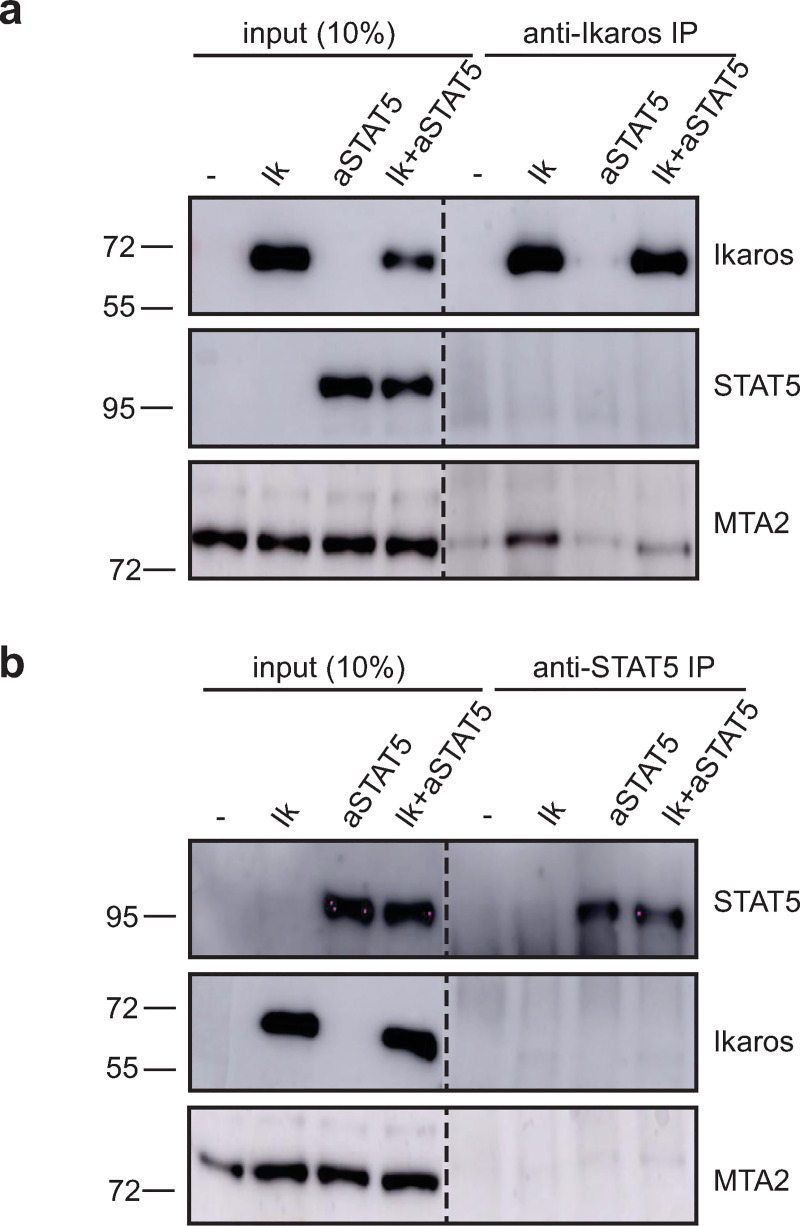
Ikaros and STAT5 do not associate. Western blot analysis for the indicated proteins after immunoprecipitation with anti-Ikaros **(a)** and anti-STAT5 **(b)** antibodies. 10% input of nuclear extracts of COS cells transfected with empty vector (-), or expression vectors for Ikaros-1 (Ik) and a constitutive active form of STAT5a (aSTAT5) are shown. The dashed lines indicate two parts of the same membrane. The original and uncropped Western blot images are provided in S1 Raw images.

To determine if Ikaros sequesters STAT5 from DNA, we evaluated the interaction of these proteins in COS cells co-transfected with Ikaros-1 and the constitutively active STAT5a protein carrying the H299R and S711F mutations (referred to as "aSTAT5"). Co-immunoprecipitation experiments were performed with nuclear extracts (Fig 5). Immunoprecipitation with anti-Ikaros antibodies brought down the known Ikaros partner MTA2, but not STAT5 (top). Conversely, immunoprecipitation with anti-STAT5 antibodies did not precipitate Ikaros (bottom). Thus, Ikaros and STAT5 do not associate in the nucleus.

### Ikaros and STAT5 target overlapping motifs at the *Cish*, *Socs2* and *Bcl6* genes

**Fig 6:**
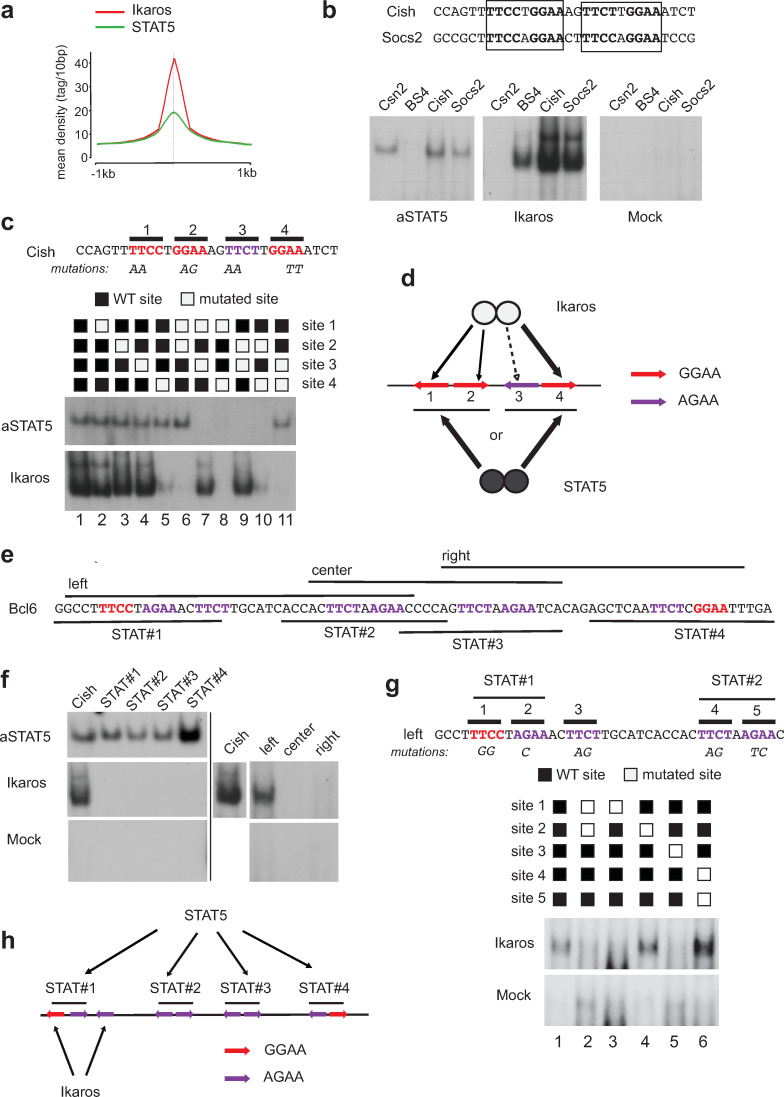
Ikaros and STAT5 target common sequences in the *Cish* and *Bcl6* genes. **(a)** Tag density profiles of Ikaros and STAT5 on the 1163 genomic regions bound by both proteins by ChIP-seq. For both proteins, the curve is centered on the summit of the Ikaros peak. **(b)** Binding of Ikaros and the constitutive STAT5a mutant (aSTAT5) by EMSA to a conserved element present under the Ikaros/STAT5 peak summits in the *Cish* and *Socs2* genes. *Csn2* and BS4 are control probes known to bind respectively STAT5 or Ikaros. Mock corresponds to nuclear extracts from COS cells transfected with an empty expression vector. **(c)** Mutational analysis of STAT5 and Ikaros binding to the *Cish* probe (EMSA). **(d)** Schematic model of the binding of STAT5 and Ikaros dimers to the *Cish* element. **(e)** Sequence of the *Bcl6* regulatory region under the summit of the STAT5 and Ikaros peaks. STAT5 target motifs are in red, and the various probes used for EMSA (in f) are indicated. **(f)** Binding of Ikaros and STAT5 to the *Bcl6* regulatory regions. **(g)** Mutational analysis of Ikaros binding to the left probe. **(h)** Schematic representation of the binding of Ikaros and STAT5 to the *Bcl6* regulatory region. The black lines represent the 4 STAT5 target sites. The original and uncropped EMSA autoradiograms are provided in S1 Raw images.

Both Ikaros and STAT5 recognize motifs that often contain the core GGAA sequence, suggesting that both proteins may compete for binding to DNA. In addition, our ChIP-seq results indicated that the summits of the Ikaros and STAT5 peaks frequently coincide (Fig 6A). To determine if Ikaros and STAT5 bind overlapping sites, we studied the known STAT5 target sequences at the *Cish* and *Socs2* genes (Fig 6B), which comprise similar tandem TTCC/TTGGAA motifs separated by 2 nucleotides (nts), by EMSA. The aSTAT5 protein bound to these motifs, as expected [26], and did so as well as it did to the canonical STAT5 site of the bovine β-casein gene *Csn2* [27]. In contrast, aSTAT5 did not bind to the synthetic, high affinity Ikaros motif BS4 [28]. Strikingly, Ikaros bound both the *Cish* and *Socs2* sequences better than it did to BS4, indicating that Ikaros can bind STAT5 target sequences with high affinity.

To further dissect the binding requirements of aSTAT5 and Ikaros, we evaluated the importance of the four potential Ikaros G/AGAA motifs (sites 1–4) on the *Cish* sequence, by mutational analyses and EMSA (Fig 6C). These results showed that aSTAT5 required two tandem motifs (either sites 1 and 2, or 3 and 4) to bind as well as it did to the WT sequence. In contrast, Ikaros strongly bound site 4 in combination with site 1 or 2 (lanes 7 and 9), weakly when site 1 was paired with site 3 (AGAA) in the absence of site 4 (lanes 5 and 10), and not at all whenever the G/AGAA motifs were adjacent to each other (lanes 6, 8 and 11). Thus, both aSTAT5 and Ikaros can bind the *Cish* sequence, but in different ways. aSTAT5 requires one complete STAT5 target sequence, while Ikaros can bind parts of distinct STAT5 target sequences but requires a space of >2 nts between GGAA motifs. These results also indicated that aSTAT5 cannot bind to its target sequence if Ikaros is present (Fig 6D).

We also investigated aSTAT5 and Ikaros binding at the *Bcl6* regulatory sequence, which comprises a cluster of 4 putative STAT5 target sequences within a 73 bp distance in exon 1 (Fig 6E). aSTAT5 bound each of these target sequences individually by EMSA (Fig 6F), but Ikaros did not, similar to our results with the *Cish* sequence. To determine if Ikaros can bind the G/AGAA motifs of neighboring STAT5 target sequences, we tested Ikaros binding on longer sequences ("left," "center" and "right"; Fig 6E and 6F). Ikaros bound only to the left probe which contained 5 G/AGAA motifs, including one (site 3) located between two canonical STAT5 target sequences. Mutational analysis of the 5 motifs revealed that Ikaros bound to sites 1 and 3, suggesting again that Ikaros partially binds the STAT5 target sequence, probably because of space requirements (Fig 6G and 6H). Thus, Ikaros targets one of the four STAT5 target sequences in the *Bcl6* locus.

Altogether, our data indicated that Ikaros antagonizes STAT5 function at the level of DNA binding at the majority of STAT5 target genes, at least in part by direct competition.

## Discussion

Here we provide a molecular explanation for why Ikaros blocks IL-7/STAT5 signaling in pre-B cells during B cell differentiation, and why Ikaros suppresses the tumorigenic effects of constitutively active STAT5 mutations in BCP-ALL [3, 13]. We show that Ikaros inhibits DNA binding by STAT5 at the great majority of STAT5 target genes, using genome-wide analyses. Further we show that Ikaros and STAT5 target overlapping DNA sequences, in two gene models (*Cish and Bcl6*), and directly compete for binding to repress or activate transcription. These results indicate that common target genes of Ikaros and STAT5 are critically sensitive to the levels of these regulators for their expression. Our data support and extend the observations of Katerndahl et al. [13], who reported that Ikaros, STAT5 and NFκB regulate common super-enhancers, and that IL-7 treatment of leukemic cells with active STAT5 reduces Ikaros binding at the *Cish* promoter.

IL-7/STAT5 signaling is important at the pro-B cell stage, and its downregulation is required for differentiation to pre-B cells [29, 30]. The switching off of the IL7/STAT5 response has been shown to be due partly to reduced cell adhesion and migration away from IL-7-rich niches [2]. Our results suggest that increasing Ikaros levels during B cell development is essential for evicting STAT5 from its target genes. Indeed, this antagonism may largely explain the tumor suppressor function of Ikaros in BCP-ALL, where *IKZF1* loss-of-function mutations are most prevalent in subtypes exhibiting activated JAK/STAT5 signaling. These results have implications beyond the B cell lineage, as STAT5 and Ikaros family proteins are also important for T cell activation and polarization downstream of IL-2 and IL-7 signaling [31–33].

Our data confirm *Bcl6* as an important gene repressed by STAT5 in pre-B cells, in agreement with previous data showing repression of *Bcl6* expression by IL-7, followed by a pre-BCR mediated activation of *Bcl6* expression during pre-B cell differentiation [34]. The strong binding of STAT5 to *Bcl6* is consistent with the presence of 4 adjacent STAT5 target sequences in the *Bcl6* first exon.

Our analysis of the *Cish* and *Bcl6* regulatory elements showed that Ikaros and STAT5 compete to bind GGAA motifs. While STAT5 binds a canonical target sequence (inverted motifs separated by 1 nt), Ikaros cannot, perhaps because the motifs are too close together. Instead Ikaros binds one GGAA motif from neighboring STAT5 target sequences, and/or a second motif nearby. Further, Ikaros binding appears to be more flexible in term of motif spacing and orientation. In our hands, Ikaros can bind tail-to-tail repeats separated by 12 nts (*Cish* gene sites 1 and 4), or direct repeats separated by 7 nts (*Cish* gene sites 2 and 4, *Bcl6* gene sites 1 and 3). In addition, the Ikaros synthetic BS4 site contains a head-to-head repeat separated by 4 nts. These results suggest that Ikaros dimers can bind GGAA motifs in a variety of configurations with high affinity, which may explain why so many STAT5 target sites are bound by Ikaros. However, binding is not identical, as STAT5 binds GGAA and AGAA well, while Ikaros favors mainly GGAA, as seen by the marginal to no binding to *Cish* site 3 and the STAT5 target sequences #2–4 at *Bcl6*. Thus, Ikaros and STAT5 may directly compete only at the GGAA motif.

Ikaros may antagonize STAT5 through additional mechanisms. Ikaros binding at common target genes is more widespread than the regions bound by STAT5. This is clearly visible at the *Bcl6* locus, where Ikaros binds to multiple sites that do not exhibit a STAT5 target sequence. Ikaros binding to "STAT5-free" sites may be important for recruiting chromatin remodeling complexes which in turn may interfere indirectly with STAT5 function [35–37], either at common targets or via other pathways such as the modulation of histone acetylation [13]. Indeed, one of the main genes repressed by Ikaros in pre-B cells is *Ptk2* which encodes focal adhesion kinase 1 [5], whose downregulation is required for increased motility and migration away from IL-7 sources [2]. Ikaros may thus act at multiple checkpoints to antagonize IL-7/STAT5 signaling, though it does not seem to affect STAT5/pSTAT5 levels.

**Fig 7:**
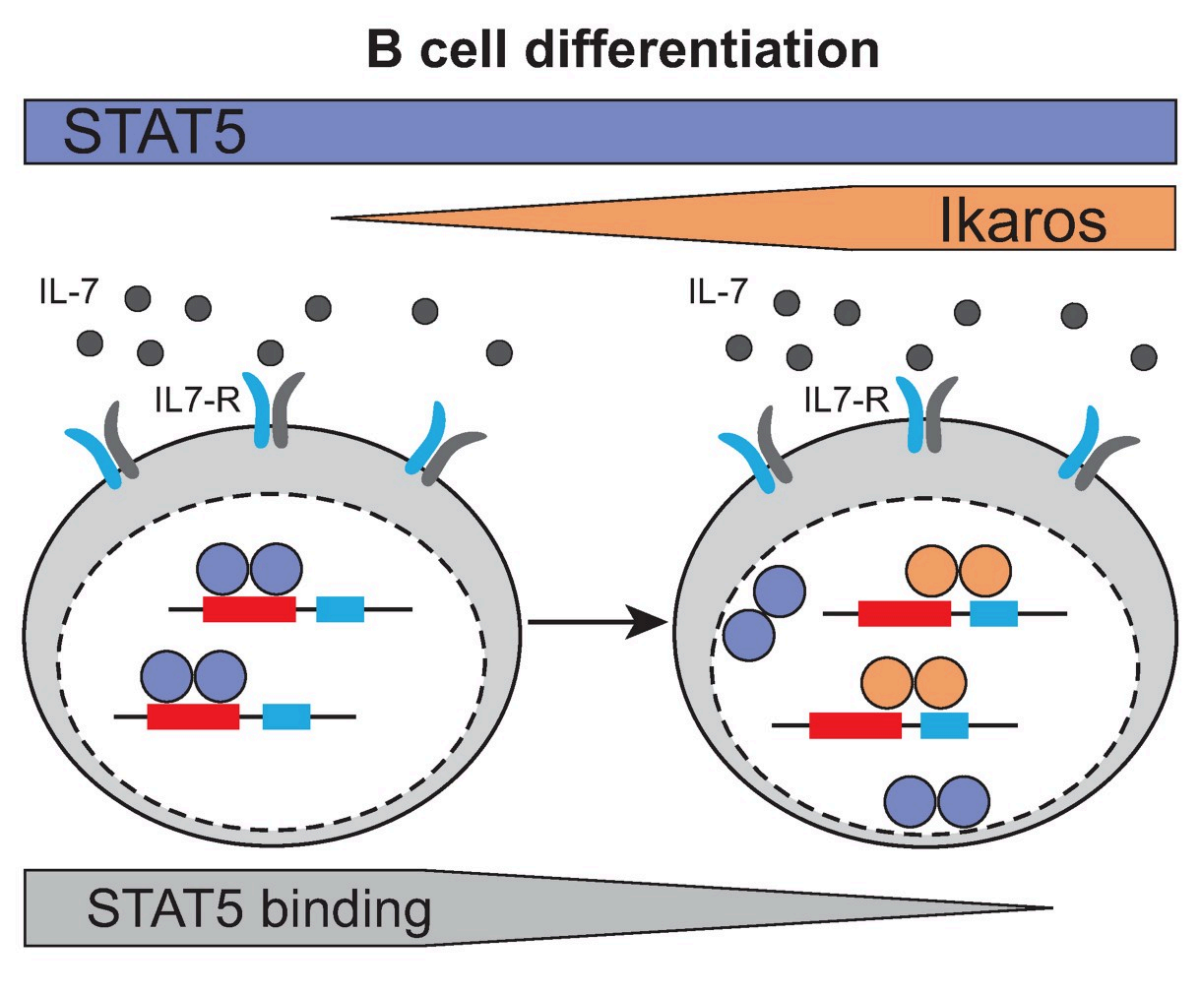
Competition of STAT5 and Ikaros binding to common target genes during B cell differentiation. During B cell differentiation, increasing Ikaros protein levels induce the displacement of STAT5 from DNA by the partial overlap of Ikaros/STAT5 binding sites at common target genes.

In conclusion, our study demonstrates that increasing Ikaros levels during B cell differentiation is important for antagonizing IL-7/STAT5 signals via direct competition for overlapping binding sites (Fig 7).

## Supporting information

S1 Fig IL-7-dependent regulation of genes with regions bound by STAT5 and not Ikaros. GSEA using as gene set genes closest to regions bound by STAT5 only (i.e. not bound by Ikaros), and as the ranked gene list all probesets present on the 430 2.0 array, ranked according to the fold change (FC) of expression between IL-7 treated and deprived cells cultured in the absence of 4OHT (24h). NES: normalized enrichment score. The p value is calculated by GSEA on the basis of 100 random permutations of the ranked gene list.
(TIF)

S2 Fig Impact of Ikaros on the gene most strongly activated by IL-7. Comparison of IL-7 dependent activation in the presence and absence of Ikaros. IL-7-dependent fold changes (IL-7 vs no IL-7) were calculated for cells cultured in the presence of vehicle (Ikaros inactive) or 4OHT (Ikaros active). The graph represents the log2 of the FC for all genes that were activated >2x by IL-7 in the absence of 4OHT, and bound by Ikaros and STAT5 at common regions. The transcriptome data are from the dataset GSE51350.
(TIF)

S1 Raw images (PDF)
