# Correction: Ikaros antagonizes DNA binding by STAT5 in pre-B cells

**DOI:** 10.1371/journal.pone.0246570

**Published:** 2021-01-29

**Authors:** Beate Heizmann, Stéphanie Le Gras, Célestine Simand, Patricia Marchal, Susan Chan, Philippe Kastner

In Fig 1, all the DNA motifs are missing from panel C. Please see the correct Fig 1 here.

**Fig 1:**
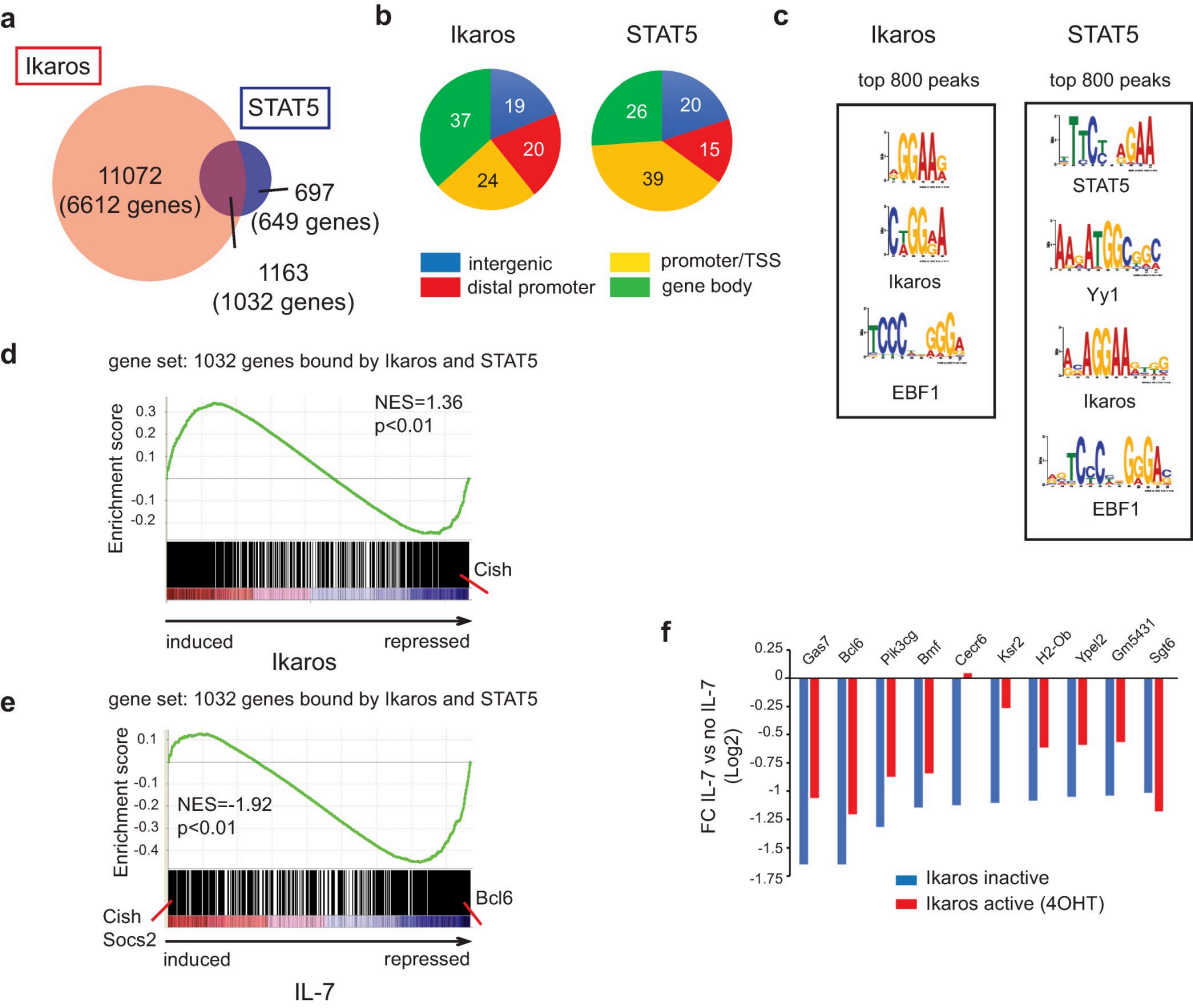
Ikaros and STAT5 DNA binding correlates with Ikaros- and IL-7-dependent gene expression. (a) Venn diagram depicting the number and overlap of Ikaros and STAT5 binding peaks. (b) Distribution of Ikaros and STAT5 binding peaks on transcriptional start site (TSS) regions (defined as located between -1kb and +100bp of the TSS), gene body (all exons and introns), distal promoter regions (located between -20kb and -1kb of the TSS) and intergenic regions (all other regions). (c) Enriched motifs among the Ikaros and STAT5 bound peaks. Enriched motifs were identified with the MEME algorithm within an 80 bp window centered on the peak summit, and significantly enriched motifs are depicted. Motif analysis was performed on the top 800 peaks [ranked by a score corresponding to nb tags x fold enrichment over input x -10log(p-value)]. (d) GSEA using as gene set genes bound at common genomic regions by both Ikaros and STAT5, and as the ranked gene list all probesets present on the 430 2.0 array, ranked according to the fold change (FC) of expression between IL-7 deprived cells cultured in the presence or absence of 4OHT (24h). (e) GSEA of the same gene set as in (d), where the gene list was ranked according to the FC of expression values measured for cells cultured with or without IL-7 (24h), in the absence of 4OHT. NES: normalized enrichment score. In (d) and (e), the p value is calculated by GSEA on the basis of 100 random permutations of the ranked gene list. (f) Comparison of IL-7-dependent repression in the presence and absence of Ikaros. Genes that were bound by both Ikaros and STAT5 at common regions, and repressed by IL-7 more than 2-fold in the absence of 4OHT, were selected. IL-7-dependent FCs (IL-7 vs no IL-7) were calculated for cells cultured in the presence of vehicle (Ikaros inactive) or 4OHT (Ikaros active). In (d), (e) and (f), transcriptome data are from the dataset GSE51350.
