# Supplementary figures and images for: Ikaros antagonizes DNA binding by STAT5 in pre-B cells
Source: PLoS One. 2020 Nov 12;15(11):e0242211. doi: 10.1371/journal.pone.0242211 (PMC7660478; doi:10.1371/journal.pone.0242211)

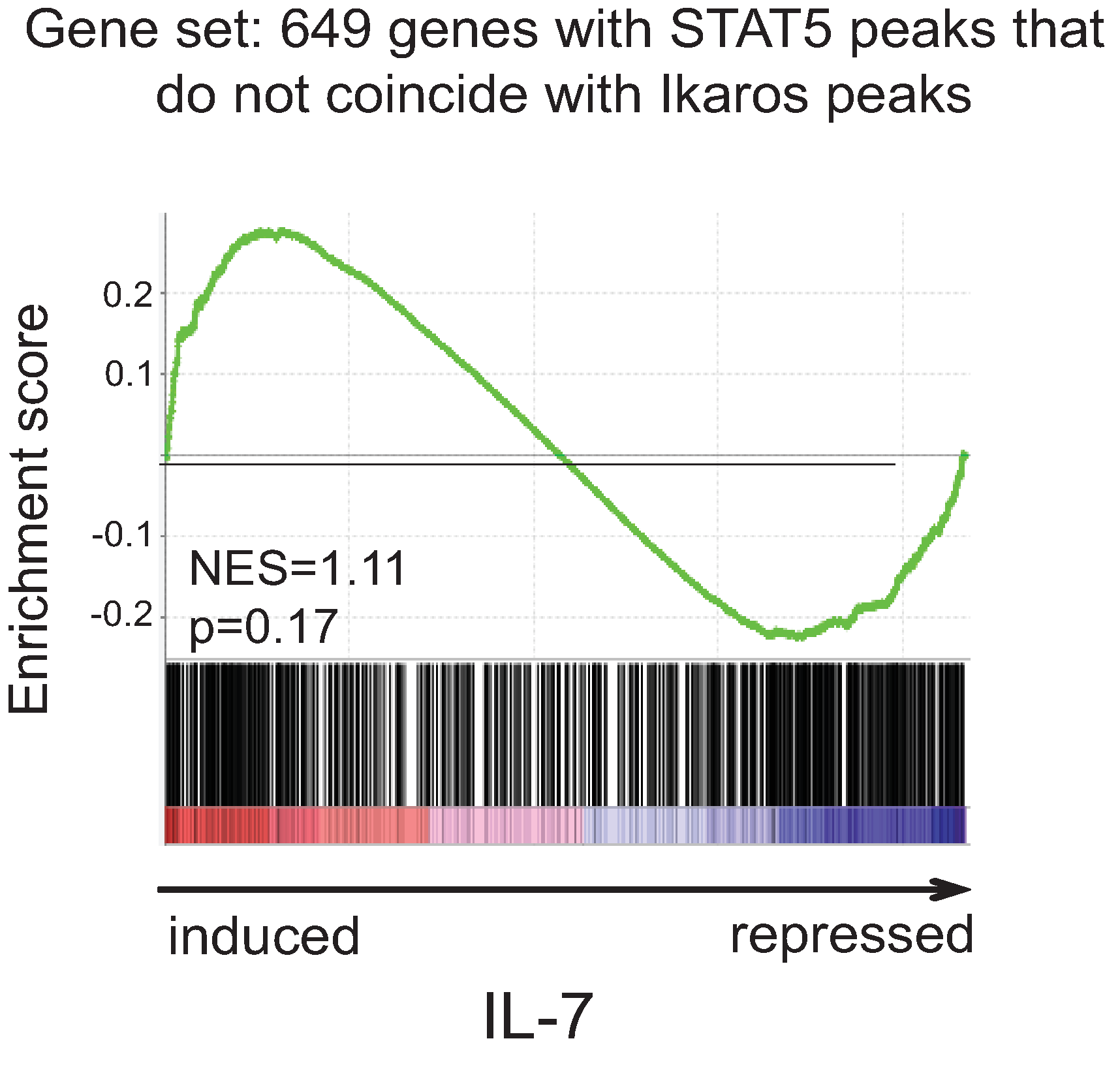

Supplement: S1 Fig — GSEA using as gene set genes closest to regions bound by STAT5 only (i.e. not bound by Ikaros), and as the ranked gene list all probesets present on the 430 2.0 array, ranked according to the fold change (FC) of expression between IL-7 treated and deprived cells cultured in the absence of 4OHT (24h). NES: normalized enrichment score. The p value is calculated by GSEA on the basis of 100 random permutations of the ranked gene list. (TIF) [file pone.0242211.s001.tif]

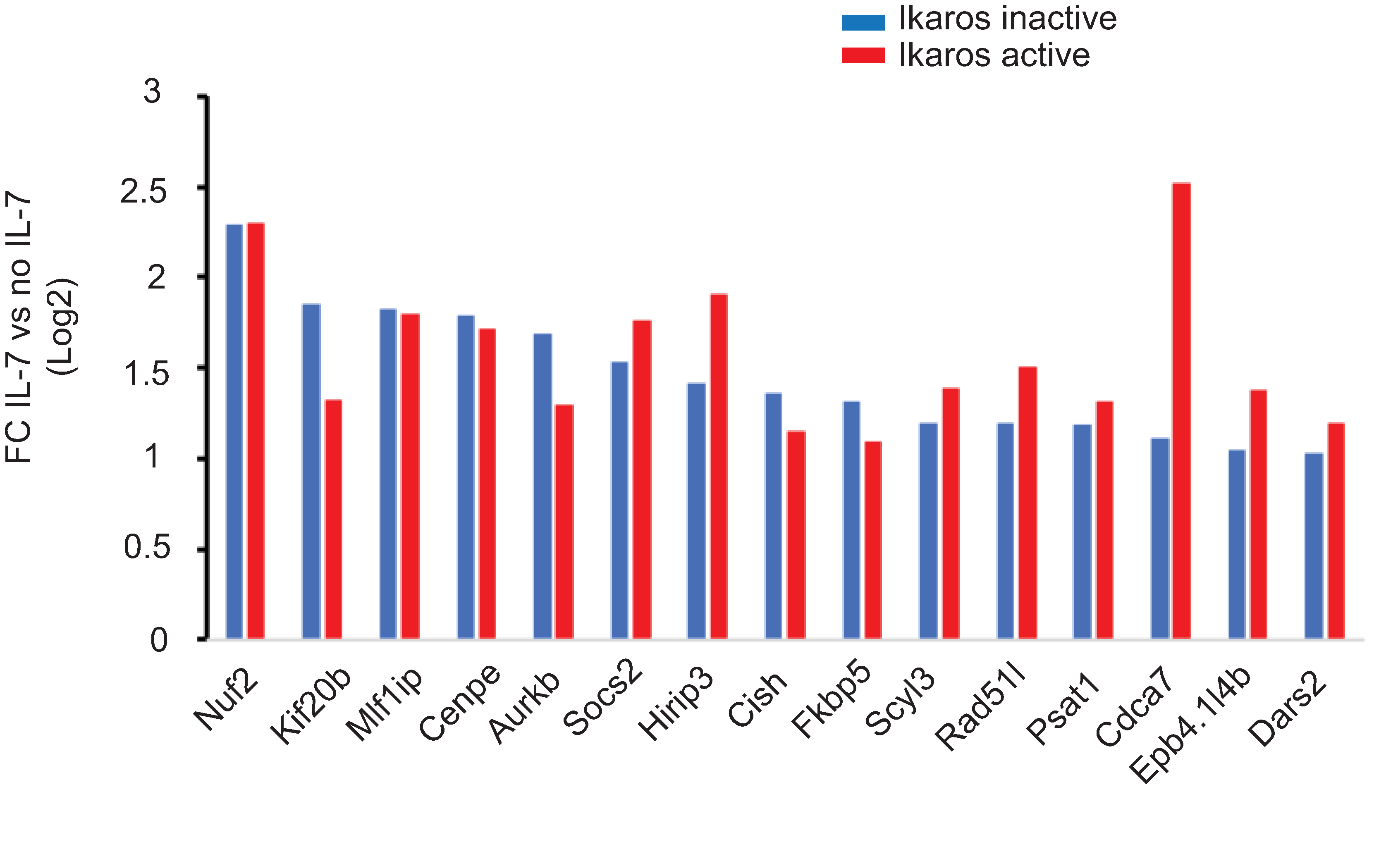

Supplement: S2 Fig — Comparison of IL-7 dependent activation in the presence and absence of Ikaros. IL-7-dependent fold changes (IL-7 vs no IL-7) were calculated for cells cultured in the presence of vehicle (Ikaros inactive) or 4OHT (Ikaros active). The graph represents the log2 of the FC for all genes that were activated >2x by IL-7 in the absence of 4OHT, and bound by Ikaros and STAT5 at common regions. The transcriptome data are from the dataset GSE51350. (TIF) [file pone.0242211.s002.tif]

Fig 5a

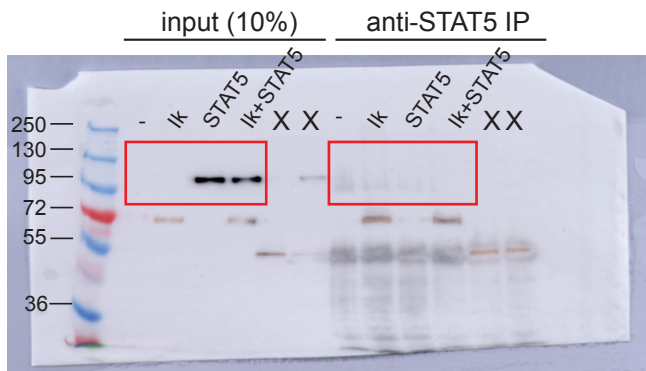

Fig 5b

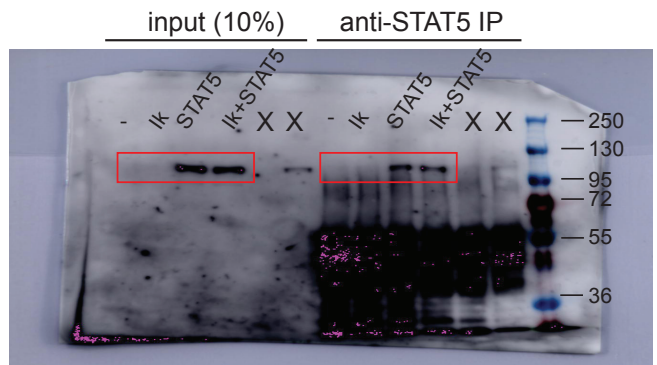

STAT5

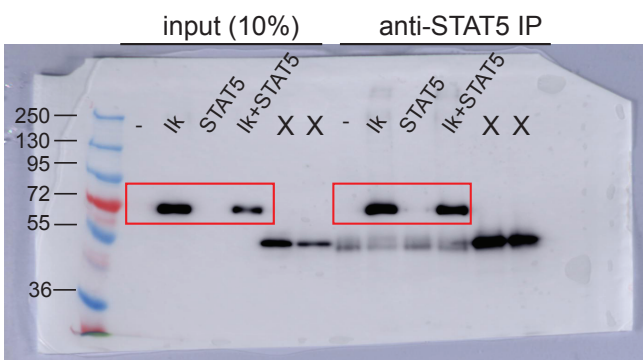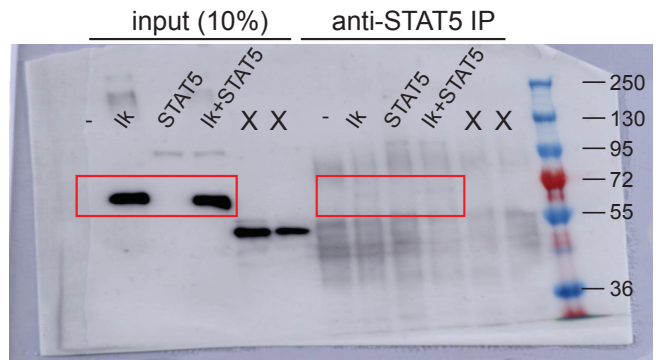

Ikaros

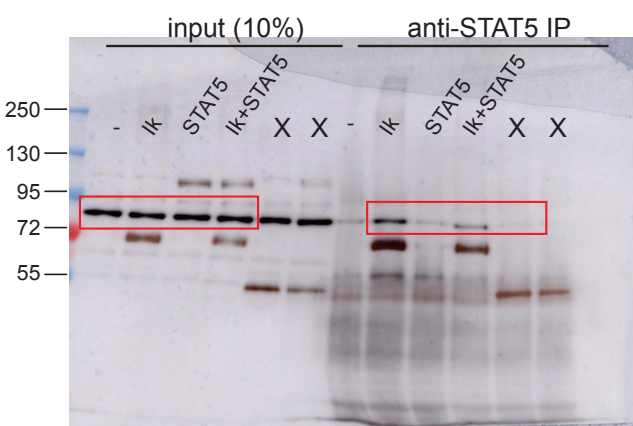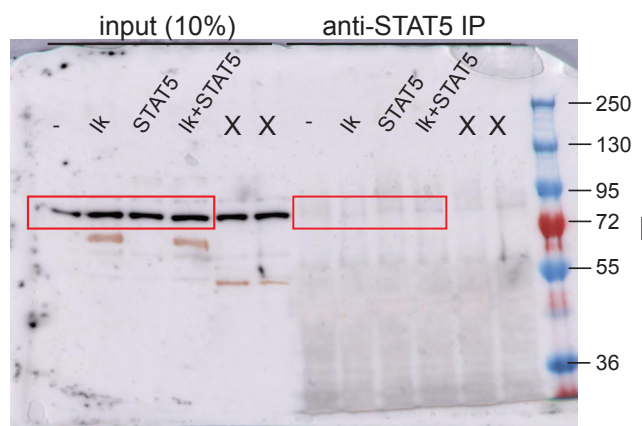

MTA2

Fig 6b

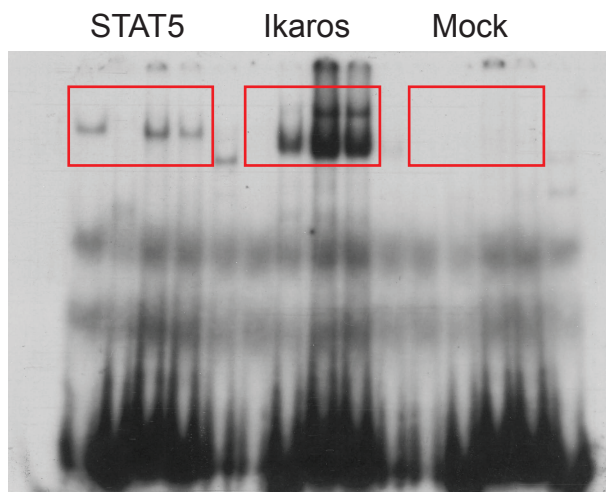

Fig 6c

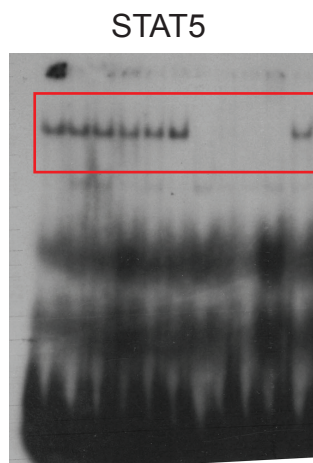

Fig 6c

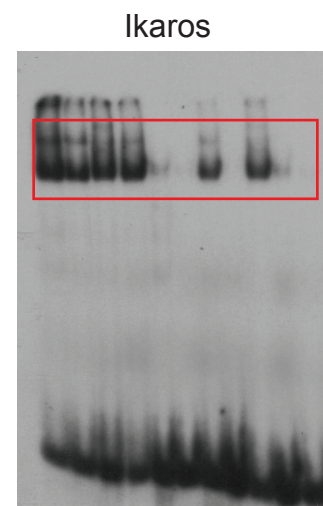

Fig 6f, left panels

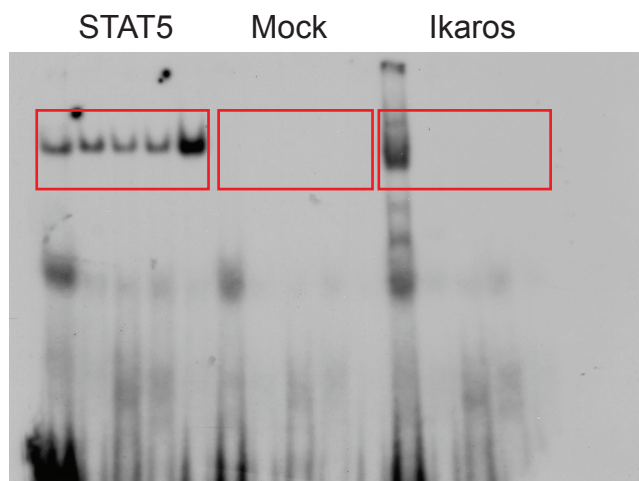

Fig 6f, right panels

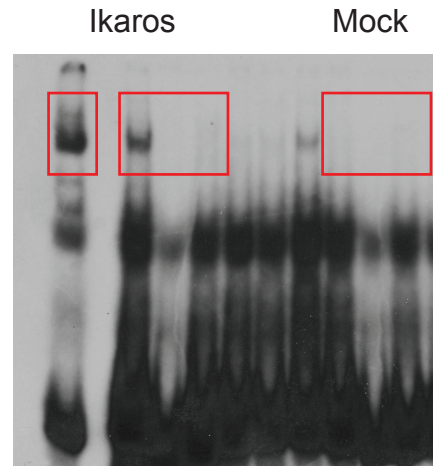

Fig 6g

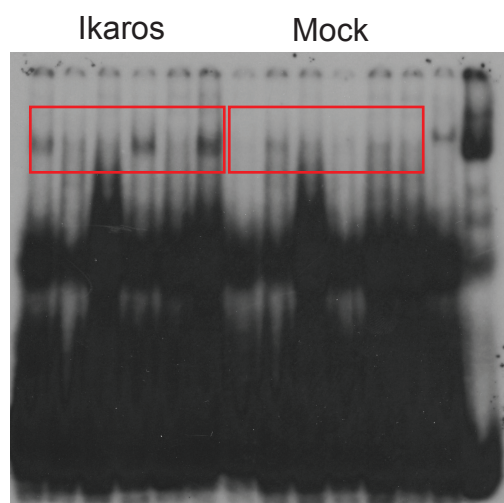

S1\_raw\_images

Supplement: S1 Raw images — (PDF) [file pone.0242211.s003.pdf]
